# Correction to Comparing the Efficacy of Plasma Exeresis and Cryotherapy for the Treatment of Seborrheic Keratosis: A Randomized Controlled Trial

**DOI:** 10.1111/srt.70312

**Published:** 2025-12-21

M. Noorbakhsh, Y. Kalantari, E. Ghasemi, B. Shokri, H. Mahmoudi, M. Daneshpazhooh, Ifa E., and M. Khani, “Comparing the Efficacy of Plasma Exeresis and Cryotherapy for the Treatment of Seborrheic Keratosis: A Randomized Controlled Trial,” *Skin Research and Technology* 29, no. 8 (2023): e13429, https://doi.org/10.1111/srt.13429.

The published article contains incomplete or inaccurate statements. The following sections have been added to supplement the original content or replace inaccurate published statements.

**Ethical Approval**

**Addendum**: This clinical study was approved by the Ethics Committee of Tehran University of Medical Sciences under approval code IR.TUMS.MEDICINE.REC.1399.573. All participants provided informed consent prior to inclusion in the study.

**Conflicts of Interest**

**Original**: None to declare.

**Corrected**: At the time of the research, co‐author Dr. Mohammadreza Khani was affiliated with Plasma Fanavar Jam Co., the manufacturer of the plasma device used in this study. The authors declare that the device was provided solely for research purposes, and there were no financial relationships, funding, or commercial interests that could have influenced the conduct or outcomes of this research.

**Data Availability Statement**

**Original**: Not Applicable.

**Corrected**: The data supporting the findings of this study, including de‐identified patient photographs and raw images underlying the figures, are available from the corresponding author upon reasonable request and in compliance with ethical and privacy considerations.

The authors apologize for these oversights and for any inconvenience they may have caused.

